# Development and Characterization of Microparticles with *Actinidia arguta* Leaves Extract by Spray-Drying: A New Mind-Set Regarding Healthy Compounds for Oral Mucositis

**DOI:** 10.3390/antiox12081496

**Published:** 2023-07-26

**Authors:** Filipa Teixeira, Ana Margarida Silva, Stefania Sut, Stefano Dall’Acqua, Cristina Delerue-Matos, Berta Estevinho, Paulo C. Costa, Francisca Rodrigues

**Affiliations:** 1 REQUIMTE/LAQV, ISEP, Polytechnic of Porto, Rua Dr. António Bernardino de Almeida, 4249-015 Porto, Portugal; 10210131@ess.ipp.pt (F.T.); ana.silva@graq.isep.ipp.pt (A.M.S.); cmm@isep.ipp.pt (C.D.-M.); 2 Department of Pharmaceutical and Pharmacological Sciences, University of Padova, Via Marzolo 5, 35121 Padova, Italy; stefania.sut@unipd.it (S.S.); stefano.dallacqua@unipd.it (S.D.); 3 LEPABE, Laboratory for Process Engineering, Environment, Biotechnology and Energy, Faculty of Engineering, University of Porto, Rua Dr. Roberto Frias, 4200-465 Porto, Portugal; berta@fe.up.pt; 4 ALiCE—Associate Laboratory in Chemical Engineering, Faculty of Engineering, University of Porto, 4099-002 Porto, Portugal; 5 REQUIMTE/UCIBIO, MedTech-Laboratory of Pharmaceutical Technology, Department of Drug Sciences, Faculty of Pharmacy, University of Porto, Rua de Jorge Viterbo Ferreira, 228, 4050-313 Porto, Portugal; pccosta@ff.up.pt; 6 Associate Laboratory i4HB—Institute for Health and Bioeconomy, Faculty of Pharmacy, University of Porto, 4050-313 Porto, Portugal

**Keywords:** spray-drying, *Actinidia arguta* leaves, microencapsulation, antioxidant compounds, oral diseases

## Abstract

*Actinidia arguta* leaves have gained notoriety over the past years due to their rich bioactive composition with human pro-healthy effects, particularly in relation to antioxidants. Nevertheless, antioxidants are well known for their chemical instability, making it necessary to develop suitable delivery systems, such as microparticles, to provide protection and ensure a controlled release. The aim of this work was to produce polymeric particles of *A. arguta* leaves extract by spray-drying that may improve the oral mucositis condition. Microparticles were characterized by size, shape, antioxidant/antiradical activities, swelling capacity, moisture content, and effect on oral cells (TR146 and HSC-3) viability, with the aim to assess their potential application in this oral condition. The results attested the microparticles’ spherical morphology and production yields of 41.43% and 36.40%, respectively, for empty and *A. arguta* leaves extract microparticles. The *A. arguta* leaves extract microparticles obtained the highest phenolic content (19.29 mg GAE/g) and antioxidant/antiradical activities (FRAP = 81.72 µmol FSE/g; DPPH = 4.90 mg TE/g), being perceived as an increase in moisture content and swelling capacity. No differences were observed between empty and loaded microparticles through FTIR analysis. Furthermore, the exposure to HSC-3 and TR146 did not lead to a viability decrease, attesting their safety for oral administration. Overall, these results highlight the significant potential of *A. arguta* leaves extract microparticles for applications in the pharmaceutical and nutraceutical industries.

## 1. Introduction

*Actinidia arguta* is a perennial vine that mostly grows in Asian countries, particularly Japan, Korea, and China [1,2]. The increased production observed in Europe and North America in recent years [3] is mainly due to the growing scientific evidence that the consumption of *A. arguta* fruit is associated with different therapeutic properties and pro-healthy benefits for consumers, particularly antioxidant, anti-inflammatory and antiviral effects [4,5,6,7]. Despite the richness of *A. arguta* fruit in bioactive compounds, its leaves have recently attracted a lot of attention, being described as a traditional herbal medicine in Korea and consumed as food [8]. These pro-healthy properties are due to the leaves’ outstanding content in bioactive compounds, particularly vitamin C (7.47 mg/g of fresh weight (fw)) [9] and phenolic compounds (599.24 mg/g of dry weight (dw)) [10], such as neochlorogenic, chlorogenic, cryptochlorogenic, gallic and protocatechuic acids, rutin, kaempferol-3-*O*-rutinoside, kaempferol-3-*O*-glucoside, isorhamnetin-3-*O*-rutinoside, epicatechin, and catechin [5,11,12,13,14]. *A. arguta* leaves have become increasingly studied due to their interesting phytochemical profile as well as their reported biologic activities, such as antioxidant, anti-inflammatory, antidiabetic, antimicrobial, and anti-allergic effects [5,8,14,15,16,17,18,19]. Recently, our research group evaluated the in vivo bioactivity and safeness of the antioxidant compounds present in an *A. arguta* leaves extract [20]. Briefly, Wistar rats (*n* = 6/group) received orally the *A. arguta* leaves extract for 7 days, attesting to a remarkable enhancement of the free radical scavenging enzymes, such as superoxide dismutase, glutathione peroxidase, and catalase [20]. This powerful antioxidant potential attracted the scientist’s attention to the beneficial effects of *A. arguta* leaves against oxidative stress, such as oral mucositis (OM), the most common debilitating complication of chemotherapy. OM is characterized by the presence of erythematous and ulcerated lesions in the oral mucosa, associated with pain. The OM first-line therapy is unsatisfactory as it results in a short duration of modest pain relief, being characterized by an increased need for opioid analgesics and parenteral/enteral nutrition [21]. Therefore, the use of bioactive compounds extracted from natural sources with biological properties may be an approach to prevent/treat OM. However, the bioactive compounds concentration absorbed in the buccal mucosa is often low, due to the saliva turnover, tongue and masticatory movements, enzymatic degradation, and lack of epithelium permeation, avoiding an acceptable therapeutic effect. To overcome these disadvantages, encapsulation could be a strategy to improve the buccal delivery. During microencapsulation, the antioxidant compounds are introduced into a matrix or a polymeric wall system that protects them from external conditions, avoiding oxidation and controlling interactions and release [22,23]. To the best of our knowledge, this is the first study that explores the microencapsulation of antioxidant compounds obtained from *A. arguta* leaves extract with the main goal to be used as a nutraceutical ingredient to prevent/treat oral oxidative diseases, such as OM.

## 2. Materials and Methods

### 2.1. Chemicals and Reagents

Almost all reagents were acquired from Sigma-Aldrich (Steinheim, Germany) and Sigma Chemical Co. (St. Louis, MO, USA). Eudragit RS 30 D was kindly supplied by Evonik Operations GmbH (Darmstadt, Germany). For the LC/DAD-ESI-MS analysis, the solvents employed were supplied by Merck (Darmstadt, Germany). The human tongue squamous carcinoma cell line (HSC-3) as well as the human squamous cell carcinoma (TR146) were provided from American Type Culture Collection (ATCC, Manassas, VA, USA). Cells reagents were supplied by Invitrogen Corporation (Life Technologies, S.A., Madrid, Spain).

### 2.2. A. arguta Leaves Extraction

*A. arguta* leaves were randomly harvested from 10 different species in October 2021 at Mini-Kiwi Farm (Landim, Vila Nova de Famalicão, Portugal). The leaves were dehydrated (Excalibur Food Dehydrator, Sacramento, CA, USA) at 41 °C for 24 h and ground in a miller (Moulinex A320). Afterward, samples were stored at 4 °C until extraction. The *A. arguta* leaves extract was prepared by ultrasound-assisted extraction (UAE) using an ultrasonic probe processor (Sonics Vibra Cell, model VCX50, Newtown, CT, USA) with a 13 mm probe, according to Silva et al. [14]. The conditions used were a solid:liquid ratio of 10% (*w/v*), for 31.11 min, an ultrasonic intensity of 30 W/m^2,^ and water as solvent. The extract was filtered through Whatman n°1 paper and stored at 4 °C until further analysis.

### 2.3. Polymeric Microparticles

Spray-drying was used to produce two different microparticles: empty microparticles, only composed by the encapsulating agent (Eudragit RS 30 D (30% w/w)), and microparticles with a core of *A. arguta* leaves extract. The technique was performed using a mini spray dryer BÜCHI B-290 (Flawil, Switzerland) with a standard 0.5-mm nozzle, according to Chaumun et al. [24]. Solutions were fed into the spray dryer at a flow rate of 4 mL/min (15%) and at an inlet temperature of 115 °C. Air pressure and aspiration rate were set to 5–6 bar and 100% (36 m^3^/h), respectively. The outlet temperature was around 55–60 °C. The solutions fed to the spray-dryer were prepared by mixing 2 mL of *A. arguta* leaves extract and 198 mL of encapsulating agent solution (3%, *v/v*), according to preliminary tests.

### 2.4. Polymeric Microparticles Characterization

#### 2.4.1. Production Yield

The production yield was calculated from the ratio between the total amount of powder collected from the device’s inner cylinder and the total amount of extract and/or excipients in the feed solution, as represented in Equation (1).

(1) $$Yield\left(\%\right)=\frac{weigth\;of\;the\;collected\;powder}{inicial\;weigth\;of\;extract/excipients\;in\;the\;feed\;solution}\times 100$$

#### 2.4.2. Encapsulation Efficiency and Loading Capacity

The encapsulation efficiency (%) was determined by the ratio between the mass of each bioactive compound present in the microparticles and the mass of each bioactive compound used to prepare the solution fed to the spray-dryer (Equation (2)), considering the production yield [25]. The loading capacity (%) was determined by the ratio between the mass of each bioactive compound and the mass of microparticles (Equation (3)).

(2) $$Encapsulation\;efficiency\left(\%\right)=\frac{mass\;of\;each\;compound\;in\;the\;microparticles}{mass\;of\;each\;compound\;in\;the\;solution\;feed\;to\;the\;spray\;-\;dryer}\times 100$$

(3) $$Loading\;capacity\left(\%\right)=\frac{mass\;of\;each\;compound\;in\;the\;microparticles}{mass\;of\;microparticles}\times 100$$

#### 2.4.3. Phenolic Profile by LC/DAD-ESI-MS

The phenolic compounds present in the *A. arguta* leaves extract, as well as in the produced microparticles, were determined by liquid chromatography equipped with triple quadruple mass spectrometry (LC/DAD-ESI-MS), according to the procedure described by Silva et al. [26]. The stationary phase used was an Agilent Eclipse XDB C-18 (3.0 × 150 mm) 3.5 μm column and the mobile phase consisted of a gradient of three compounds: 1% formic acid (A), acetonitrile (B), and methanol (C). The gradient started at 95% A, 5% B, and 0% C and continued to 0% A, 90% B, and 10% C, over 30 min. The flow rate was 0.4 mL/min, with the column temperature stable at 30 °C. The sample injection volume was 20 µL. Both produced microparticles and the *A. arguta* leaves extract were individually diluted 10 times in methanol:water (50:50, *v:v*) and centrifuged at 13,300 rpm for 15 min. MS spectra were recorded in negative ion mode in the 99–1200 *m*/*z* range. For quantification purposes, the standard calibration curves used were:

Chlorogenic acid curve: y = 198.01x + 20.138 (*R*^2^ = 1)

Rutin curve: y = 58.564x + 41.752 (*R*^2^ = 0.9998)

The results obtained were expressed as μg of each phenolic compound per mL of sample (μg phenolic compound/mL sample).

#### 2.4.4. Particle Shape, Structure, and Surface Properties

Scanning electron microscopy (SEM) was employed to investigate the polymeric microparticle morphology, using a Fei Quanta 400 FEG ESEM/EDAX Pegasus X4M (Eindhoven, The Netherlands). These analyses were carried out in a JEOL JFC-100 apparatus with an accelerating voltage of 15 kV and a magnification of 100–50,000 X. Samples were placed in the analyzer by fixating the microparticles in a brass stub with double-sided adhesive tape and then coated in a vacuum by a thin layer of gold.

#### 2.4.5. Particles Size and Distribution

The particles’ diameter was studied by using a Mastersizer™ 3000 laser diffraction particle size analyzer equipped with Malvern’s Hydro EV dispersion unit (Malvern Instruments; Worcestershire, UK). Microparticles were placed on the dispersion liquid (distilled water) with a concentration corresponding to laser light obscuration between 5 and 10%. Before reading, the particles were left in the dispersion unit under rotation (2000 rpm) for 10 min and 1 min under ultrasonication (50%) to stabilize the solution and remove aggregates. The material type was selected as non-spherical, with a refractive index of 1.5, an absorption index of 0.01, and a density of 1. The number of readings was 5 consecutive measurements, 10 sec each for both background and sample. The mean size over volume, the percentiles 10, 50, and 90 of the equivalent spherical diameters based on volume (Dv10, 50, and 90), and the span are given by the Mastersizer’s software (version 3.63, Malvern Instruments; Worcestershire, UK) and were considered for microparticles characterization.

#### 2.4.6. Rheological Behavior

The rheological behavior of both empty and loaded microparticles was analyzed in a rotational rheometer (Malvern Kinexus Lab+, Malvern Instruments, Worcestershire, UK) with a plate-plate geometry (PU20 SR4367 SS) presenting a diameter of 20 mm and a gap of 1 mm, at 25 °C. The microparticles were individually dispersed in distilled water and then placed in the loading chamber for further measurements. The start and end frequency applied were 10 and 0.1000 Hz, respectively, with a shear strain of 0.25% (value in the Linear Viscoelastic Region). The analysis was completed in triplicate, and the viscosity data (Pa.s) were collected and processed using the rSpace Kinexus Lab+ software (version 1.75, Malvern Instruments, Worcestershire, UK).

#### 2.4.7. Total Phenolic Content

The Folin–Ciocalteu method was used to determine the total phenolic content (TPC), following the methodology described by Singleton and Rossi [27], with minor modifications. Gallic acid was used as a standard curve (linearity range = 5–100 µg/mL; *R^2^* > 0.998), and the results were expressed as mg of gallic acid equivalents (GAE) per gram of microparticles (mg GAE/g).

#### 2.4.8. DPPH Radical Scavenging Activity Assay

DPPH free radical scavenging assay was performed following the procedure described by Barros et al. [28], with minor modifications. Trolox was used as standard solution (linearity range = 5–125 µg/mL; *R^2^* > 0.998). Results were expressed as mg of Trolox equivalents (TE) per gram of microparticles (mg TE/g).

#### 2.4.9. Ferric Reducing Antioxidant Power (FRAP) Assay

The FRAP assay was performed according to Benzie & Strain [29], with minor modifications. Ferrous sulfate heptahydrate (FeSO_4_·7H_2_O) was used as a standard solution (linearity range = 25–500 µM; *R*^2^ > 0.996). The results were expressed in µmol of ferrous sulphate equivalents (FSE) per gram of microparticles (mg FSE/g).

#### 2.4.10. Moisture and Swelling Capacity

The microparticles moisture content and swelling capacity were evaluated by determining the water content before and after hydration, according to Campos et al. [30], with minor modifications. Firstly, 150 mg of dry microparticles were weighed and placed in an infrared moisture balance AD-4713 (A&D Company; Tokyo, Japan), where an infrared lamp heated the particles at 103 °C for 60 min. The device automatically calculated the moisture percentage through the differential of the sample weight before and after heating. Afterward, new microparticles were hydrated for 4 h with PBS (pH 7.4) and gently collected by filtration with Whatman nº 1 paper to remove the excess of water. The increase in water mass was determined as the indirect swelling ability of the microparticles.

#### 2.4.11. Differential Scanning Calorimeter (DSC)

Differential Scanning Calorimeter (DSC) experiments were performed individually on the formulated microparticles and on the lyophilized leaves extract, through a DSC 200 F3 Maia (Netzsh–Gerätebau GmbH, Germany), using the following setting parameters: temperature from 0 °C to 200 °C and heating rate of 10 °C/min. Samples were sealed in an aluminum pan with a perforated lid, using the following setting parameters: temperature from 0 °C to 200 °C and heating rate of 10 °C/min. The onset temperatures were calculated using Proteus Analysis software (Version 6.1, Netzsh-Gerätebau GmbH, Germany).

#### 2.4.12. Fourier-Transform Infrared Spectroscopy (FTIR)

The interactions between the polymer used and the extract encapsulated were evaluated using a FTIR Nicolet 6700—diamond point (ThermoFisher Scientific, Waltham, MA, USA) and the disc of potassiumbromide (KBr) method. The microparticles powders as well as all the excipients used were individually placed in the sampler with a spectra analysis between 4000 and 400 cm^−1^ and 32 scans at a resolution of 4 cm^−1^.

#### 2.4.13. In vitro Biocompatibility Studies

The biocompatibility of the unloaded and loaded microparticles was assessed by cell viability using the 3-(4,5-dimethylthiazol-2-yl)-2,5-diphenyltetrazolium bromide (MTT) colorimetric assay. TR146 (human buccal cell line; passage 31–32) and HSC-3 (human tongue squamous carcinoma cell line; passage 23–24) were grown in Dulbecco’s Modified Eagle Medium (DMEM), previously supplemented with 10% Fetal Bovine Serum, 1% essential amino acids, and 1% antibiotic. The cells were maintained at a temperature of 37 °C with 5% CO_2_, and the culture medium was changed every two days until it reached confluence. In each well of a 96-well plate, the cells were seeded at a concentration of 25 × 10^3^ cells/well and incubated for 48 h at 37 °C with 5% CO_2_ to provide exponential growth. After the incubation period, the medium was removed, incubated with different concentrations of loaded and unloaded microparticles (62.5–1000 μg/mL), and dissolved in the respective medium, for 24 h. Afterwards, the MTT was added to each well and incubated for 3 h at 37 °C with 5% of CO_2_. At the end, the optical density measured at a wavelength of 570 and 630 nm (SynergyTM HT Multi-mode microplate reader, BioTek Instruments Inc.; Winooski, VT, USA). The DMEM culture medium was used as a positive control and Triton X-100 1% (% *w*/*v*) as a negative control. The cell viability (CV) was calculated considering the control group, untreated, as 100% (Equation (4)):

(4) $$CV\left(\%\right)=\frac{A_{570}-A_{630}\;treat\;cell}{A_{570}-A_{630}\;untreat\;cell}\times 100$$

### 2.5. Statistical Analysis

All measurements were performed in triplicate and the results were presented as mean ± standard deviation (SD) of at least three independent assays. A value of *p* < 0.05 was considered significant after the one-way analysis of variance (ANOVA), through the IBM SPSS Statistics 27.0 software (SPSS Inc., Chicago, IL, USA).

## 3. Results and Discussion

### 3.1. Production Yield

One of the most important factors during encapsulation by spray-drying is the production yield [31], which is defined as the quantity of powder recovered, considering the quantities of raw materials and encapsulating agents used. In the present study, the production yields obtained for the empty microparticles and the microparticles with *A. arguta* leaves extract were, respectively, 41.43% and 36.40%, being lower than 50%. These results were expected due to the small quantity of raw material employed when compared to the spray dryer scale. Furthermore, some losses occur throughout the equipment [31,32], mainly due to the powder adhesion to the equipment walls as well as to the cyclone low efficiency to separate small particles, with their possible aspiration by the vacuum system [31,33]. The same phenomenon has been verified by other authors. For example, Ribeiro et al. [34] attested a lower product yield for microparticles with elderberry extract (25%) than for empty microparticles (41%), while Gonçalves et al. [35] encapsulated vitamin A in coconut oil with gum arabic and achieved a product yield of 25%, significantly lower than the result for the empty microparticles (70%).

### 3.2. Encapsulation Efficiency and Loading Capacity

**Figure 1:**
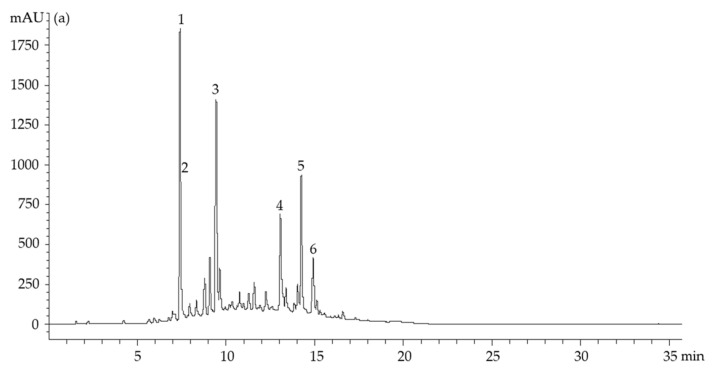

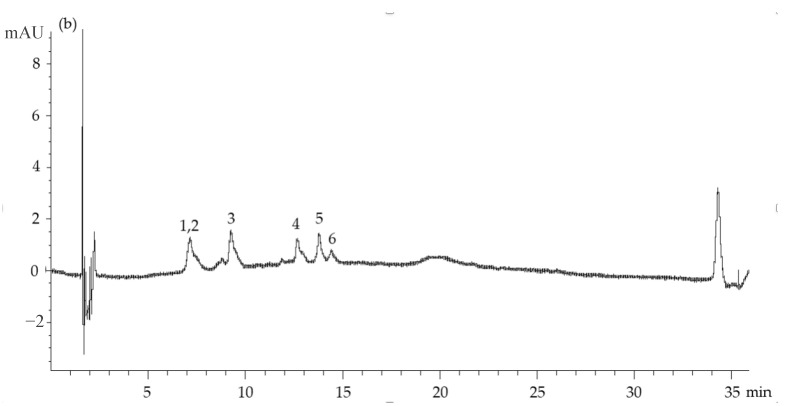
LC/DAD-ESI-MS chronograms of *A. arguta* leaves extract (**a**) and microparticles with *A. arguta* leaves extract (**b**); peak identification: (1) 1-*O*-caffeoylquinic acid, (2) 3-*O*-caffeoylquinic acid, (3) 4-*O*-caffeoylquinic acid, (4) quercetin-3-*O*-glucoside, (5) kaempferol-3-*O*-(acetyl-rhamnoside)-hexoside, and (6) kaempferol-3-*O*-(acetyl-rhamnoside)-hexoside 2.

The compounds present in loaded microparticles and *A. arguta* leaves extract were identified and quantified by LC/DAD-ESI-MS. Figure 1 shows the obtained spectra.

A total of six compounds were quantified in the extract (Figure 1a), kaempferol-3-*O*-(acetyl-rhamnoside)-hexoside being the most abundant one (180.74 μg/mL), followed by 1-*O*-caffeoylquinic acid (157.76 μg/mL), quercetin-3-*O*-glucoside (134.38 μg/mL), 4-*O*-caffeoylquinic acid (109.94 μg/mL), kaempferol-3-*O*-(acetyl-rhamnoside)-hexoside 2 (91.20 μg/mL), and 3-*O*-caffeoylquinic acid (27.45 μg/mL). Concerning the *A. arguta* leaves extract microparticles, the same compounds were identified (Figure 1b). The amounts varied from 0.09 μg/mL to 0.64 μg/mL for 3-*O*-caffeoylquinic acid and kaempferol-3-*O*-(acetyl-rhamnoside)-hexoside, respectively. These results are in line with Silva et al.’s [14] description of the phenolic composition of *A. arguta* leaves extracted by UAE, which demonstrated the presence of various chlorogenic acids and quercetin derivatives, with a particular predominance of 1-*O*-caffeoylquinic acid, 3-*O*-caffeoylquinic acid, and 4-*O*-caffeoylquinic acids.

Based on these results, it was possible to calculate the encapsulation efficacy and the loading capacity of *A. arguta* leaves extract microparticles (Table 1). The encapsulation efficiency ranged between 13.65 and 21.88% for 1-*O*-caffeoylquinic acid and kaempferol-3-*O*-(acetyl-rhamnoside)-hexoside 2, respectively. Kaempferol-3-*O*-(acetyl-rhamnoside)-hexoside and kaempferol-3-*O*-(acetyl-rhamnoside)-hexoside 2 share the same m/z and most of the fragmentation. Based on the spectra and the literature assigned, the compounds are derivatives of kaempferol-3-*O*-rhamnoside, both presenting acetylation and a further unit of six carbon-bearing sugar (hexose). The differences can be in the position of linkage of the acetylation and of the second sugar as well as different hexoses, such as glucose or galactose.

Concerning the loading capacity, the kaempferol-3-*O*-(acetyl-rhamnoside)-hexoside showed the highest value (0.00320%), followed by 4-*O*-caffeoylquinic acid (0.00225%) and 1-*O*-caffeoylquinic acid (0.00205%). These parameters are crucial to evaluate the encapsulation efficiency that expresses the amount of extract entrapped in microparticles [32,36]. Many conditions such as compounds solubility in the encapsulating agent or in the aqueous phase, aggregation or precipitation of molecules, or even spray-drying conditions (e.g., flow rate, inlet, and outlet temperature) can affect the encapsulation efficiency [36,37,38]. In the present study, even though the spray-drying conditions were adjusted to prevent the phenolic compounds’ degradation, it is possible that adjustments should be optimized. Furthermore, the ability of a compound to form molecular bonds with water molecules, which depends on certain characteristics like polarity, water solubility, and molecular size, can significantly impact the encapsulation behavior [38]. Therefore, compounds with a high affinity for water tend to lead to lower encapsulation efficiencies [37] since they rapidly form bonds with water molecules upon contact with moisture, leading to their dissolution or dispersion in the surrounding aqueous medium.

### 3.3. Surface Morphology

**Figure 2:**
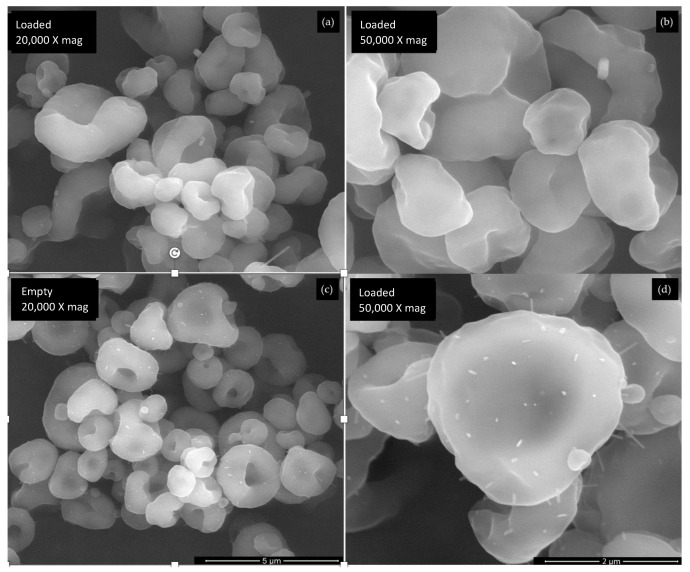
SEM images of (**a**,**b**) microparticles with *A. arguta* leaves extract and (**c**,**d**) empty microparticles. Magnification of 20,000× and 50,000×, and beam intensity (HV) of 15 kV.

**Figure 3:**
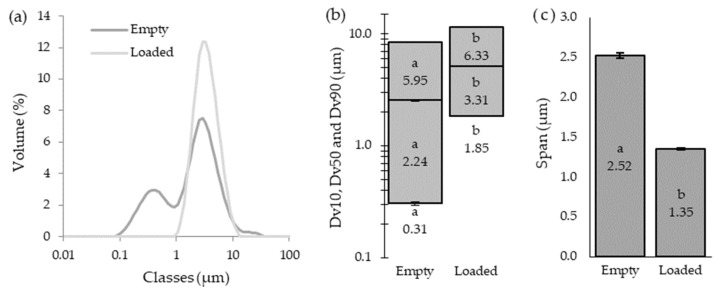
Study of the empty and loaded microparticle (**a**) size distribution over volume, (**b**) distribution graphic (Dv10 (bottom of the rectangles), Dv50 (middle lines), and Dv90 (top of the rectangles)), and (**c**) span. Data are expressed as mean ± SD (*n* = 3). Different alphabetic characters stand for a different statistical subset (*p* < 0.05) within each parameter.

The SEM analysis is presented in Figure 2. The similarities in drying conditions and the encapsulating agent for both formulations suggest that the morphological differences between microparticles are primarily due to the core material. Both microparticles display a spherical morphology, with a heterogeneous size distribution, although the *A. arguta* leaves extract microparticles present a slightly smoother surface. These findings indicate that the core material has a minimal impact on the microparticles’ morphology, which is in line with the last observations reported [24,31]. In terms of shape, both microparticles exhibit a biconcave disc-like form. This characteristic can be due to the encapsulating agent employed that has more prominent influences on the microparticles’ shape due to their intrinsic properties [24]. Specifically, the selected encapsulating agent used, namely Eudragit RS 30 D, influences the external appearance of the microparticles by creating a “bumpy” or “doughnut-like” appearance [33,39,40]. Furthermore, the analysis disclosed some agglomerates in the empty microparticles, as confirmed by the evaluation of the particle size that revealed two peaks in the volume distribution curves (Figure 3a).

### 3.4. Microparticles’ Size

The microparticles’ size distribution can be influenced by the core materials and the encapsulating agents’ properties as well as by the drying conditions, concentration, and viscosity of the encapsulated material [24,31,41]. The size distribution by volume (Figure 3a) of the empty and loaded microparticles were significantly different (*p* < 0.05), with the first ones displaying a lower mean size (2.90 µm vs. 3.75 µm, respectively). The distribution curve of the loaded microparticles followed normal behavior, which is in accordance with the lower span value (1.35 µm) achieved (Figure 3c). The span value indicates how wide or narrow the range of particle sizes are within a sample; therefore, a smaller span value is more desirable, since it indicates a narrow particle size distribution [41]. Conversely, the empty microparticles showed a distribution curve with two peaks. This bimodal distribution could result from collisions between the droplets formed in the spray-drying nozzle or between partially or completely dried particles that, in turn, lead to agglomeration [24]. Furthermore, microparticle agglomerations were observed in SEM images (Figure 2c,d) as well as with a significantly higher span value (2.52 µm), which supports this theory.

In order to characterize the size distribution of the produced microparticles, the Dv10, Dv50, and Dv90, were evaluated (Figure 3b). The empty microparticles showed Dv10, Dv50, and Dv90 values of 0.31 µm, 2.24 µm, and 5.95 µm, respectively, while the loaded ones achieved results of 1.85 µm, 3.31 µm, and 6.33 µm, respectively. Based on these results, it is possible to affirm that the loaded microparticles are larger than the empty ones in the lower size range (Dv10: 1.85 µm vs. 0.31 µm), in the median particle size (Dv50: 3.31 µm vs. 2.24 µm), and in the upper size range (Dv90: 6.33 µm vs. 5.95 µm). Size is an important characteristic for nutraceutical applications, since microparticles must not be visually or orally detected to be appealing to consumers. As for the pharmaceutical industry, the microparticles’ sizes need to be carefully controlled, since small ones may pass into the bloodstream and penetrate cells, causing DNA damage [24]. On the other hand, large microparticles can lead to the clogging of blood vessels [24]. In addition, microparticle size is a key aspect of the efficiency and stability of the encapsulated compounds, dose uniformity, sedimentation rate, and even for patient acceptance [24,42].

### 3.5. Rheological Behavior

**Figure 4:**
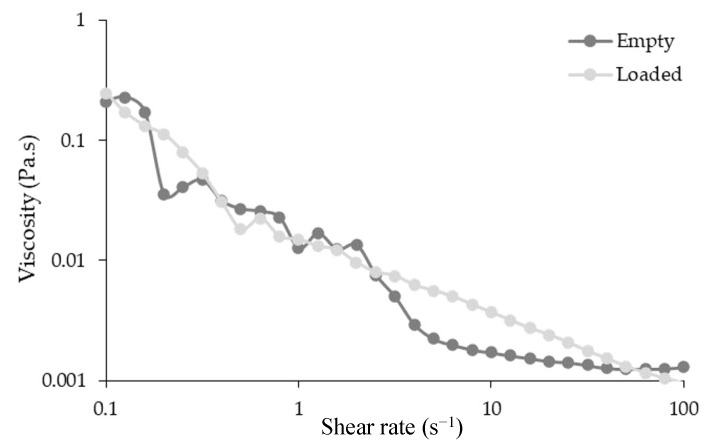
Rheological behavior of empty and loaded microparticles. Results are expressed as mean ± standard deviation (*n* = 3).

The rheological analysis results are presented in Figure 4. It is notorious that microparticles exhibit pseudoplastic behavior. The loaded microparticles demonstrated a slightly higher viscosity than the empty ones, which may be related to the high moisture content. On the other hand, the presence of larger microparticles, which increase the flow resistance, as well as the influence of the molecular weights of the bioactive compounds present inside, may contribute to the loaded microparticles’ increased viscosity observed [32]. This parameter is also correlated with the production yield, since microparticles with higher viscosities tend to have lower yields as the droplets formed are stickier and easily retained in the spray dryer [31,32]. The produced microparticles with an extremely low viscosity range (0.2–0.001) may be the reason for the nonlinearity of the shear rate curves observed. As the shear rate rises, the viscosity of both samples lower until they overlap at the highest shear rates (100 s^−1^). This behavior is typically associated with pseudoplastic materials [32].

### 3.6. Total Phenolic Content and Antioxidant/Antiradical Activity

The TPC, FRAP, and DPPH assays results are summarized in Table 2. As can be observed, the TPC of the *A. arguta* leaves extract microparticles was higher than the empty microparticles (19.29 vs. 6.54 mg GAE/g). Similar findings were obtained for the FRAP assay, with the highest result being achieved by the *A. arguta* leaves extract microparticles (81.72 µmol FSE/g), significantly higher (*p* < 0.05) than the empty microparticles (28.42 µmol FSE/g). Finally, the *A. arguta* leaves extract microparticles achieved a significantly (*p* < 0.05) higher result (4.90 mg TE/g) in the DPPH assay compared with the empty microparticles (2.48 mg TE/g). The strongest antioxidant and antiradical properties observed for the loaded microparticles are due to the presence of the phenolic compounds reported above [43]. These results are in line with other authors. For example, Medina-Torres et al. [44] microencapsulated *Litsea glaucescens* by spray-drying and produced microparticles with a higher TPC (20.22 mg GAE/g) and DPPH radical scavenging capacity (IC_50_ = 1.3 mg/mL) than empty ones.

The main goal of encapsulation is to entrap bioactive compounds present in the extract inside microparticles with the aim to protect them from external factors that may lead to their deterioration [34]. Therefore, it was expected that the microparticles containing the *A. arguta* leaves extract exhibit a lower TPC and antioxidant/antiradical activity than the extract alone. Silva et al. [14] previously assessed the extract composition and reported a TPC value of 97.50 mg GAE/g dw, while the FRAP assay revealed a value equal to 1154.10 µmol FSE/g dw. In contrast to the present study, it was possible to calculate the mean inhibitory concentration (IC_50_) for the DPPH radical, reporting a value of 547.34 µg/mL. The lower activity of the loaded microparticles when compared to the extract is a positive observation since it indicates that the microencapsulation process was successful. As intended, the spray-drier encapsulated the bioactive compounds inside the microparticles, protecting them from external aggressions by reducing their reactivity [45]. Therefore, less activity was observed for the loaded microparticles when compared to the extract.

### 3.7. Hydration Ability

The moisture content and the swelling capacity of microparticles were evaluated to analyze the impact on the powder’s flowability [46]. As shown in Table 3, no significant differences (*p* > 0.05) were observed between empty and loaded microparticles for either moisture content (4.48% and 7.34%, respectively) or swelling capacity (79.96% and 90.57%, respectively). Therefore, the extract did not affect the microparticles’ hydration ability. However, considering that the moisture content may have an impact on the product yield, the loaded microparticles lower the production yield. The higher water content may lead to the formation of stickier microparticles, increasing the adhesion to the spray dryer glass walls and, therefore, reducing the product yield [32]. Furthermore, higher moisture content is undesirable since it might promote the growth of unfavorable bacteria and reduce the product’s shelf life [30,46].

### 3.8. Differential Scanning Calorimeter (DSC)

**Figure 5:**
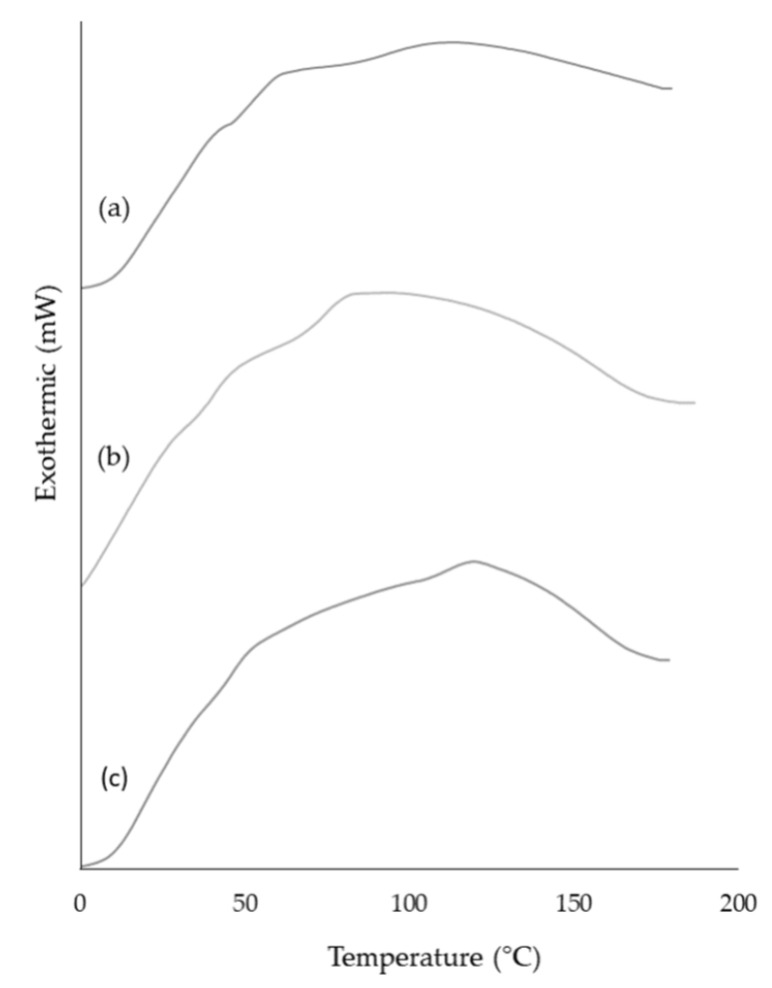
DSC thermograms of *A. arguta* leaves extract (a), empty microparticles (b), and microparticles with *A. arguta* leaves extract (c).

In order to investigate the thermal properties and stability of the produced microparticles, DSC analysis was performed in *A. arguta* leaves extract, along with empty and loaded microparticles. As shown in Figure 5, both microparticles as well as the leaves extract exhibit similar endothermic behavior, displaying no peaks in the thermograms. These results suggest that neither microparticles nor leaves extract undergo any significant thermal events within the temperature range investigated. Although not significant, it is noticeable that all samples absorbed energy (endothermic process) until around 100 °C. This occurrence may be connected to the residual water loss and molecular affinities between water molecules and samples [47]. The maximum intensity was attained at different times for the loaded and empty microparticles (Figure 5b,c), indicating that the physicochemical properties of the compounds present in the *A. arguta* leaves extract were preserved after production. The DSC analysis supported that the thermal properties are very similar for both microparticles, confirming that the stress conditions during the spray drying process did not affect the thermal properties of the microparticles’ structural components [48].

### 3.9. Fourier-Transform Infrared Spectroscopy (FTIR)

**Figure 6:**
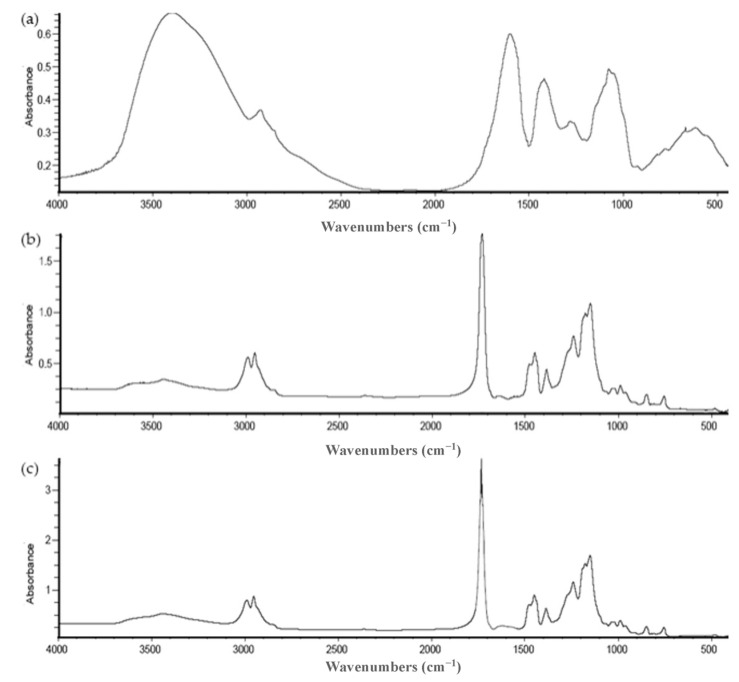
Comparative FTIR spectrum between (**a**) *A. arguta* leaves extract, (**b**) empty microparticles, and (**c**) microparticles with *A. arguta* leaves extract.

FTIR spectrometry analysis reveals the chemical properties and interactions between the molecules that constitute the compounds of interest [47]. As can be seen in the FTIR spectrum of *A. arguta* leaves extract (Figure 6a), the typical absorption bands are in the range of 500–1600 cm^−1^ and can be considered as its molecular fingerprint. Still, the presence of bands at 3400 cm^−1^ (OH groups) and 2900 cm^−1^ (C–H stretching band) should be emphasized. It is evident that the signal wavelengths 650 cm^−1^ (=C–H alkenes), 1020 cm^−1^ (C–O alcohols), 1300 cm^−1^ (C–O alcohols), 1440 cm^−1^ (C–H alkanes), and 1600 cm^−1^ (C=C alkenes) are closely related to aromatic compounds as these types of chemical bonds are the main ones present in polyphenols, such as phenolic acids and flavonoids [49]. Additionally, similar absorptions have been reported in studies where the presence of phenolic compounds from fruits and other herbal plant parts were evaluated [44,50,51].

Regarding empty and loaded microparticles, the results are represented in Figure 6b,c, respectively. In both FTIR spectra, the absence of an absorption band at λ = 3300 cm^−1^, which is characteristic of the hydroxyl group (-OH), indicates the successful drying process, confirming the absence of water. Concerning empty microparticles (Figure 6b), the obtained spectrum displayed significant peaks at wavelengths 1100–1200 cm^−1^ (quaternary ammonium group), 1700–1750 cm^−1^ (C=O stretching band related to the ester carbonyl groups), 1400–1500 cm^−1^ (C=C stretching band), and 2900–3000 cm^−1^ (C–H stretching band). Eudragit RS 30 D, widely employed in pharmaceutical formulations, serves as enteric coating material and matrix for controlled-release drug delivery systems [36]. This copolymer primarily consists of ethyl acrylate and methyl methacrylate monomers, combined with a quaternary ammonium group integrated into its structure [52]. Based on the polymer’s chemical composition, the observed peaks in the spectrum are consistent and logical. Moreover, these bands have been documented in previous studies investigating Eudragit RS 30 D using FTIR spectroscopy [33,52], supporting the claim that the spectrum obtained in the present study may accurately represent the characteristic spectrum of this polymer. For the loaded microparticles (Figure 6c), the spectrum obtained is practically the same as the empty microparticles. The typical absorption bands of the *A. arguta* leaves extract are practically lost in this spectrum, probably hidden by the absorption bands of the encapsulating polymer, indicating the success of the encapsulation process.

### 3.10. Biocompatibility Studies

**Figure 7:**
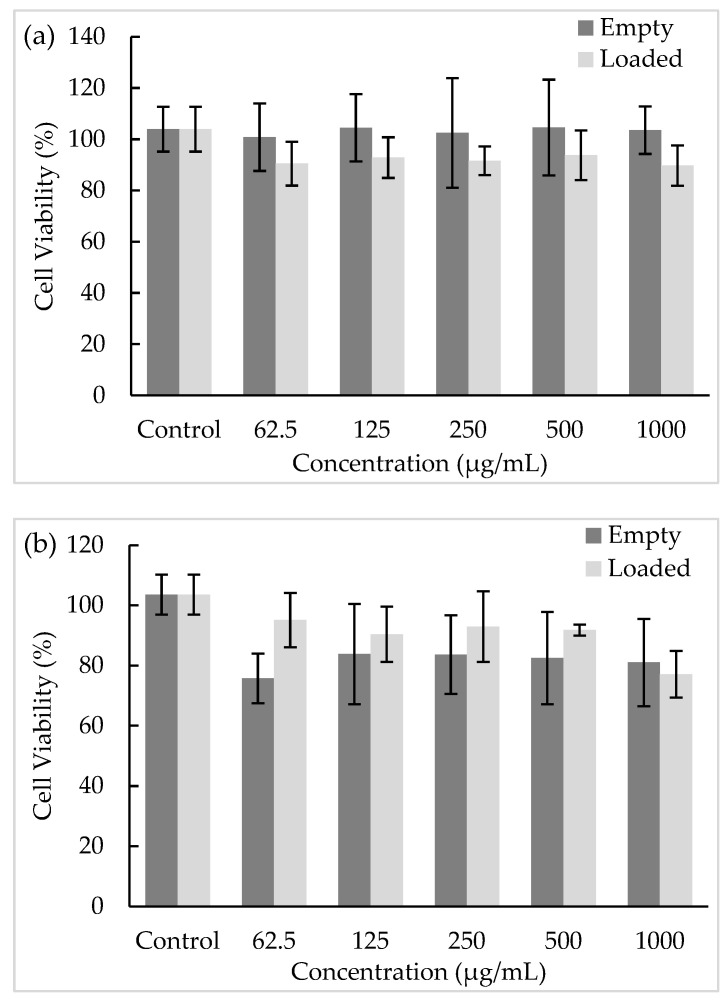
Effect of formulated loaded and unloaded microparticles on the viability of (**a**) TR146 and (**b**) HSC-3 cell lines measured by MTT assay (*n* = 3), at different concentrations (62.5–1000 µg/mL).

In vitro assays are an accurate, fast, and reproducible method to evaluate the effect of bioactive compounds in living cells [14]. In this work, the microparticles’ safety was assessed by an MTT assay in HSC-3 and TR146 cell lines, which were selected as oral cell models to evaluate the potential effects on the oral cavity (Figure 7). HSC-3 is a non-keratinized cell line derived from human tongue, while TR146 are keratinized cells derived from human buccal mucosa [53].

According to Figure 7, the empty and loaded microparticles did not affect the viability of either cell line at any of the concentrations tested, presenting viabilities between 70% and 100%. Concerning empty microparticles, their effect on TR146 cells led to viability values around 100%, while for the HSC-3 cells, the values ranged between 75.75% and 81.04% after exposure to concentrations of 62.5 and 1000 µg/mL, respectively, without significant differences (*p* > 0.05). Regarding the microparticles with *A. arguta* leaves extract, the exposure to TR146 cells led to a viability of 89.76%, while the HSC-3 viability was 77.09% at the highest concentration tested (1000 µg/mL), without significant differences (*p* > 0.05). Considering the results achieved and the oral cavity structure, these results emphasize the non-cytotoxic effect of the produced microparticles for oral administration. For example, kaempferol is described as having poor oral bioavailability, and the development of new matrix or polymeric wall systems can be a good alternative to improve bioavailability [54].

## 4. Conclusions

In the present study, the spray-drying method was employed to obtain microparticles with antioxidant compounds from *A. arguta* leaves extract. The thermal and chemical results demonstrated that the encapsulation process was successfully performed. All microparticles presented a smooth surface, spherical morphology, and heterogeneous size distribution. The microparticles’ size analysis proved the small size of the microparticles, being the lower span value of 1.35 µm. The loaded microparticles revealed the best results for antioxidant/antiradical assays, highlighting kaempferol-3-*O*-(acetyl-rhamnoside)-hexoside and 1-*O*-caffeoylquinic acid as the predominant compounds. The oral epithelia cells’ viability was not affected for both microparticles. This work showed the potential of microparticles containing *A. arguta* leaves extracts to be applied in the nutraceutical or pharmaceutical field, using a versatile and low-cost microencapsulation technique. Further studies should focus on the analysis of the compounds’ release profile through 3D cellular in vitro models as well as stability studies.

## Figures and Tables

**Table 1:** Encapsulation efficiency and loading capacity of the compounds present in the microparticles with *A. arguta* leaves extract. Results are expressed as mean ± standard deviation (*n* = 3). Different letters (a, b, c) in the same column mean significant differences (*p* < 0.05) between compounds.

	Encapsulation Efficiency (%)	Loading Capacity (%)
1-*O*-caffeoylquinic acid (1CQA)	13.65 ± 0.68 ^b^	0.00205 ± 0.00010 ^b^
3-*O*-caffeoylquinic acid (3CQA)	17.22 ± 1.72 ^a,b^	0.00045 ± 0.00002 ^c^
4-*O*-caffeoylquinic acid (4CQA)	21.50 ± 2.15 ^a^	0.00225 ± 0.00011 ^b^
Quercetin-3-*O*-glucoside	15.63 ± 1.56 ^b^	0.00200 ± 0.00010 ^b^
Kaempferol-3-*O*-(acetyl-rhamnoside)-hexoside	18.60 ± 1.86 ^a,b^	0.00320 ± 0.00016 ^a^
Kaempferol-3-*O*-(acetyl-rhamnoside)-hexoside 2	21.88 ± 2.19 ^a^	0.00190 ± 0.00010 ^b^

**Table 2:** Total phenolic content (TPC) and antioxidant/antiradical activity (the DPPH radical scavenging capacity and the ferric reducing antioxidant power (FRAP) assay, respectively) produced microparticles. Results are expressed as mean ± standard deviation (*n* = 3). Different letters (a, b) in the same column mean significant differences (*p* < 0.05) between the empty and loaded microparticles.

	TPC (mg GAE/g)	FRAP (µmol FSE/g)	DPPH (mg TE/g)
Empty microparticles	6.54 ± 0.77 ^b^	28.43 ± 3.72 ^b^	2.48 ± 0.40 ^b^
Loaded microparticles	19.29 ± 1.27 ^a^	81.72 ± 4.31 ^a^	4.90 ± 0.60 ^a^

GAE, Gallic acid equivalents; FSE, Ferrous sulphate equivalents; TE, Trolox equivalents.

**Table 3:** Moisture content and swelling capacity of produced microparticles, in percentage. Results are expressed as mean ± standard deviation (*n* = 3).

	Moisture Content (%)	Swelling Capacity (%)
Empty microparticles	4.48 ± 1.70	76.96 ± 10.62
Loaded microparticles	7.34 ± 0.47	90.57 ± 9.44

## Data Availability

The data used to support the findings of this study can be made available by the corresponding author upon request.
